# Occipital Neuralgia: a noninvasive therapeutic approach

**DOI:** 10.1590/1518-8345.2621.3067

**Published:** 2018-11-14

**Authors:** Pablo Jesús López-Soto, José Miguel Bretones-García, Verónica Arroyo-García, Margarita García-Ruiz, Eduardo Sánchez-Ossorio, María Aurora Rodríguez-Borrego

**Affiliations:** 1 Instituto Maimonides de Investigación Biomédica de Córdoba, Grupo Cuidados enfermeros integrales, Perspectiva multidisciplinar, Córdoba, Andalucía, Spain.; 2 Universidad de Córdoba, Departamento de Enfermería, Córdoba, Andalucía, Spain.; 3 Hospital Universitario Reina Sofía, Córdoba, Andalucía, Spain.; 4 Centro de Ergodinámica Córdoba, Córdoba, Andalucía, Spain.; 5 Centro Ergodinámica Barcelona, Barcelona, Cataluña, Spain.

**Keywords:** Neuralgia, Therapeutics, Foot Orthoses, Gait, Pain, Pain Management

## Abstract

**Objective:**

to evaluate the application of a noninvasive intervention consisting of a postural modification using personalized models and osteopathy in people with occipital neuralgia.

**Method:**

retrospective study of the intervention performed in adult population with occipital neuralgia, consisting of postural modification using personalized plantar orthoses and osteopathy, in a study period of four years. The observed variables were: persistence of headache, alignment of the axes, plantar support, center of gravity and center of mass; medical interview data, visual analogue scale, Win-Track gait analysis system and Kinovea software for video analysis (clinical assessment instruments used).

**Results:**

a total of 34 records of people with occipital neuralgia were studied. A fraction of 58.8% of the patients reported improvement after the intervention. The visual analogue scale data were provided for 64.7% of the records and significant differences (p <0.001) between the means before (8.4 ± 1.7) and after the intervention (2.6 ± 2.7) were found.

**Conclusion:**

postural modification using personalized orthoses and osteopathy substantially improves the symptomatology of patients with occipital neuralgia.

## Introduction

Neuralgia is considered the most frequent neuropathy. It was described by Beruto and Lentijo and Ramos in 1821, being defined as a disabling alteration characterized by recurrent headaches located in the occipital region^1^. According to the classification by the International Headache Society (IHS), occipital neuralgia (ON) is defined as “unilateral or bilateral paroxysmal pain, of lancinating or acute nature that is located in the posterior part of the scalp in the distribution of the major, minor and third occipital nerves, which on certain occasions is accompanied by reduced sensitivity or dysesthesia in the affected area and is usually associated to hypersensitivity of the affected nerve or nerves”^2^. This type of neuralgia is classified into a subset that includes post-traumatic pain, lash, cervical spine deformity, tension headache, chronic daily headache and migraine^3^ ^-^ ^4^. In addition to occipital neuralgia, the scientific community employs other terms for its definition, such as C2 neuralgia, Arnold neuralgia, or occipital neuritis^5^ ^-^ ^6^. Other characteristics are the presence of pain that recovers the ocipucius that diminishes after the use of anesthetic blocks of the affected nerves^2^ ^,^ ^4^.

ON is a multiple-cause clinical condition. In its manifestation, several factors^7^ may be involved: traumatic factors (fracture, hematoma, iatrogenic), anatomical factors (Chiari malformation, pinching, compression sensitivity), fibromyalgia, tumors (osteochondroma, neuroma, multiple myeloma, atlantoaxial lateral masses) infections (pyomyositis, neurosyphilis, pachymeningitis) and degenerative changes (atlantoaxial lateral osteoarthritis, C1-C2 arthrosis syndrome).

The therapeutic approach is very broad. Regarding treatment, the conservative methods were: use of antiepileptics, antidepressants, nervous block, non-steroidal analgesics, opioids, neuromodulators, and transcutaneous electrical nerve stimulation, as well as cervical orthoses. However, the scientific community also approves the use of surgery as the last therapeutic option: neurolysis and decompression, neurectomy, rhizotomy and ganglionectomy, C1-C2 fusion, radiofrequency ablation, and peripheral nerve stimulation^7^. Nevertheless, the use of different therapeutic options does not always result in the remission of the symptomatology, together with the fact that they are painful. Therefore, it is considered necessary to propose therapeutic alternatives that lead to more effective therapeutic results.

The starting hypothesis of the present study was the fact that the appearance of the symptoms associated with occipital neuralgia was of biomechanical origin, considering the cause of the occipital neuralgia a postural alteration that has as consequence the nervous/venous compression of the root of the C2 vertebra^8^. In this sense, we describe the results that the research group obtained with the application of customized insoles in patients with ON, obtaining an improvement in the symptomatology of ON with the postural correction of standing/walking position, as well as osteopathy. Therefore, the objective of the study is to evaluate the application of a noninvasive intervention consisting of a postural modification using personalized insoles and osteopathy in people with occipital neuralgia (ON).

## Methods

This is a retrospective observational study of pre/post-intervention records, which includes three periods of clinical evaluation (after 15 days of intervention, after 40 weeks and after one year). The objects of study were the clinical records of people older than 14 years who voluntarily sought a biomechanical clinical unit in southern Spain in the period between May 2012 and May 2016, with signs of ON.

We included records related to occipital neuralgia (previously diagnosed by a specialist), of persons older than 14 years and corresponding to the study period, in which there was a noninvasive intervention on the symptomatology.

In the clinical evaluation, the following variables were collected: socio-demographic characteristics (age and gender), clinical characteristics (years of persistence of neuralgia) and clinical-assistance characteristics. The latter variables were determined by the ergodynamic gait study [axis alignment, plantar support, center of gravity and center of mass (N/cm^2^)], posturology study [center of gravity and center of mass (N/cm^2^) and dysmetria of the lower limbs, as well as the level of pain (using the Visual Analogue Scale - VAS).

The noninvasive therapeutic intervention consisted in performing a postural modification of the affected person using plantar orthoses and osteopathy.

Subsequent to the application of the treatment, data were collected on the persistence of headache and whether some type of complementary medication was necessary, and it was advised, according to clinical criteria, some modification of the plantar orthoses. The treatment follow-up was performed in three observations (consultations at 15 days, 40 weeks and approximately one year from the start of treatment), in which the following tests were repeated in order to assess the efficacy of the intervention: ergodynamic gait study, posturology study and lower limb dysmetria; as well as post-intervention pain level.

As indicated, in addition to the medical interview for data collection, VAS was used to determine pain level, and baropodographies (N/cm^2^) were used to determine the different pressures exerted by the foot, using the NOVEL-EMED-Model AT^®^ device. As a gait analysis system, the Win-Track device (Medicapteurs, France-USA)^9^ ^)^ was used.

Finally, to carefully measure the postural axes, computer software Kinovea video analysis was used. This software draws a line from the external auditory canal to the caudal line through a normal axis. This line must coincide with distinct anatomical points (acromion-clavicular, trochanter and external-malleolus). The software measures the separation with each of certain points of the medial line, noting if there is any abnormal axis (kyphotic or lordotic).

The clinical-assistance variables were described by numerical values and percentages, and the differences in mean values and percentages were calculated by the Pearson test χ² and Fisher’s test, according to the type of variable. For comparison of means of independent groups, the Student’s t-test or the Mann Whitney U-test were used, according to normal or non-normal parameters. According to the type of variable, statistical tests of hypothesis contrast were performed, assuming a statistical significance of p <0.5 and confidence intervals with 95% confidence.

The study was carried out in agreement with the institution’s ethical guidelines and with the Helsinki Declaration, and with approval of the Research Ethics Committee (Registration No. 3281), abiding to the Organic Law 15/1999 on Personal Data Protection of the Spanish State.

## Results

Thirty-four adult individuals with episodes of ON were treated with the noninvasive intervention. The mean age of people whose records were included in the study (N = 31) was 34.1 ± 10.9 years. There was a greater proportion of women (67.6%) than men. An important percentage (35.5%) had suffered for more than 10 years with neuralgia; and in 10 patients (29.4%) there was no record of this variable. In the other registries, it was documented that the duration of the condition was 5-6 years (11.8%), 2-3 years (11.8%), and from months to one year, only three people (8.8%). Of the 34 patients studied, 17 (50%) reported taking previous medications to treat neuralgia, with anti-inflammatories being the most used type of medication (64.7%).

The ergodynamic gait study, posturology study and lower limb dysmetria evaluation were performed in all patients. Nevertheless, there are records of only one third of the subjects included in the study (N = 11, 32.3%).

**Figure 1:**
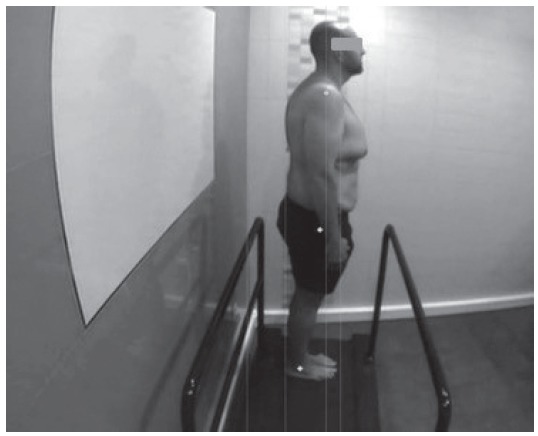
Pre-intervention posturology study. Cordoba, Andalusia, Spain, 2012-2016

Based on the reference points (acromioclavicular, major trochanter and peroneal malleolus), the established vertical did not correspond in 5 of the 11 subjects (45.4%). Besides the approximation to the reference points, the alignment between these points is also important, being the same the percentage of people (45.4%) who did not comply with this criterion, as shown in Figure 1.

**Figure 2:**
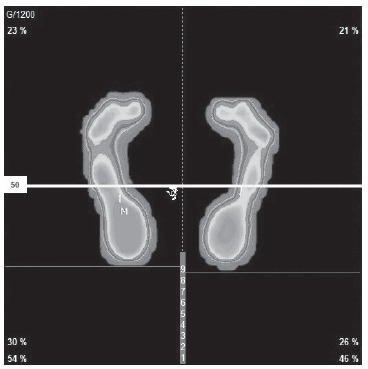
Static baropodography prior to intervention. Cordoba, Andalusia, Spain, 2012-2016

**Figure 3:**
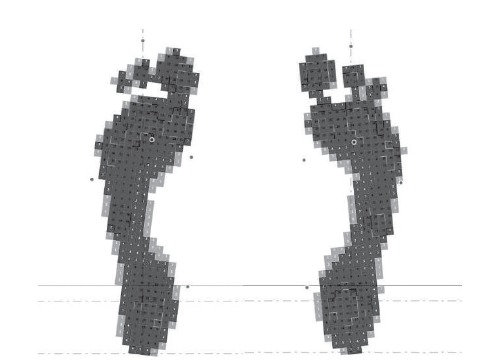
Dynamic baropodography of left and right foot before the intervention. Cordoba, Andalusia, Spain, 2012-2016

Plantar orthoses were prescribed to all patients in the study. However, osteopathy was performed in only 15 subjects (44.1%). The type of prescribed orthosis depended on the person’s type of plantar support, center of gravity and center of mass, as shown in Figures 2 and 3.

As previously indicated, 2-3 follow-up visits were performed to know the evolution of the patients and potential improvement of their condition. The first control was performed in a time frame with a median after the intervention of 64.0 days (interquartile range - RI: 47.0-72.75), in 24 subjects (82.3%). At this visit, a high percentage of patients presented a favorable evolution (79.1% of the patients evaluated, only one of them was asymptomatic). In the other patients, it was necessary to manipulate with osteopathy or to place a loop in one of the limbs due to dysmetria. The next consultation (N = 27), scheduled for the sixth month after the intervention, was performed in a median time frame of 230.0 days (RI: 201.0-284.0). The symptomatology at this visit had disappeared in five of the subjects studied (18.5% of those who attended) and was favorable in the same percentage. In this second consultation, in 44.1% of the total patients studied, no data was obtained. On the other hand, four patients (14.8%) required changes in the insoles; three decided not to continue with the insoles and presented recurrence of symptoms; and in two other subjects, the discomfort continued. The proposed consultation at 18 months (N=19) occurred after a period whose median was 608 days (RI: 546-885). Six of the patients reported being asymptomatic (31.5%). Although five of those who participated in the consultation (26.3%) reported that symptomatology had appeared, then the orthoses were modified. Another third of those who attended (36.8%) reported stability in symptomatology and one patient reported not agreeing with the treatment provided in the approach. At the last visit, with a wide variation in time, since many subjects did not require a high number of medical visits (N = 29), the period was a median of 1,312 days (RI: 409-1608.5) (43.1 month/3.6 years).

After the three follow-up visits, a large percentage of patients (N=13; 38.2%) reported no persistence of occipital neuralgia; 20.6% reported improvement and neuralgia was less persistent. However, five patients (14.7%) indicated that the symptomatology persisted and from 23.5% data was not obtained. Concerning VAS, only 64.7% (N=22) provided information on the records, with significant differences (p <0.001) between means previous (2.6 ± 2.7) and after (8.4 ± 1.7) the intervention.

At the end of the study period, 20 of the 34 subjects studied (58.8%) remained with plantar orthoses and four of them did not use them (11.7%); of the other subjects, no data was obtained. On the other hand, 35.2% of the patients did not take any medication at the end of the study; 11.7% only took it when they had a crisis and 14.7% took anti-inflammatory drugs; regarding the other subjects studied, there is no data available.

**Figure 4:**
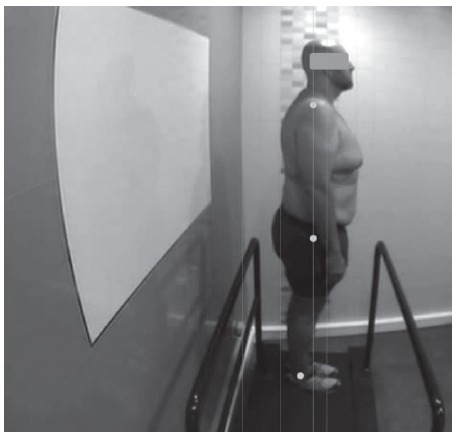
Posturology study using plantar orthoses. Cordoba, Andalusia, Spain, 2012-2016

**Figure 5:**
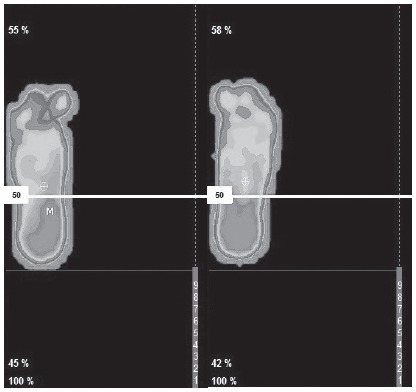
Static baropodography employing plantar orthoses. Left image: Left foot; Right: Right foot. Cordoba, Andalusia, Spain, 2012-2016

Regarding the posturology study (N = 9), including the plantar orthosis, all the subjects presented improvement in the alignment of the axis, as well as the distance to said axis, according to Figure 4. Moreover, baropodographies with insoles showed improvement in the person’s plantar support, center of gravity and center of mass, according to Figure 5.

## Discussion

ON has been a well-established entity for many years, but there is no consensus on the intervention to be performed as treatment. Usually, most are invasive conservative interventions and noninvasive measures are poorly considered. Nevertheless, the present study provides information about the effectiveness of postural correction as an ON treatment with plantar orthosis, and when the clinician considered it appropriate, osteopathy.

The most common therapeutic measure is the infiltration of local anesthetic agents with and without steroids, a technique that alleviates pain in some cases (15% -36%)^10^ ^-^ ^12^. In this sense, a high percentage of patients (58.8%) experienced pain reduction after orthoses implantation. In addition to infiltration with or without steroids, botulinum toxin A was used for its inhibitory effect on the motor plaque as a muscle relaxant, although its possible analgesic action was indirect^13^ ^-^ ^14^, reducing only the associated acute and pungent ON pain, not constant and only pain for several months^15^. The postural correction intervention showed continuous improvement in the three follow-up visits, a fact that strengthens the application of these measures in future studies.

On the other hand, some studies show the efficacy of pulsed radiofrequency in the treatment of ON. Nevertheless, the known studies are observational without controls and in the same way as in the previous case, only short and medium-term pain control is observed^16^ ^-^ ^18^.

Surgical techniques are also used for the treatment of ON, such as occipital neurolysis and stimulation of the occipital nerve. However, it has been documented that the use of these techniques increases the possibilities of developing entities of worse therapeutic control than neuralgia, for example, neuroma or regional pain syndrome^19^.

However, the present study has several limitations. Despite the fact that there were statistically significant differences after postural correction in ON symptomatology, the retrospective nature and consequently, follow-up losses, does not allow to determine exclusively the effectiveness of the treatment, due to the fact that there are no control groups. Moreover, the VAS scale determines the perception of pain, nevertheless, the control of neuropathic pain requires evaluation of the psychological aspects^20^, a fact that is not usually considered in interventional studies in this neuralgia.

## Conclusion

The application of customized orthoses, and in some cases osteopathy, substantially improves the postural alignment (acromion-clavicular, trochanter and external malleolus) and as a consequence, the symptomatology of ON. It is possible to conclude that after the noninvasive intervention, the level of neuropathic pain decreased significantly.

This therapeutic alternative, according to our knowledge, was not approached by the scientific community and could be considered as a first approach in the treatment of ON. On the other hand, clinicians should consider that invasive and/or surgical techniques may trigger less controllable clinical conditions than the underlying entity. In this sense, a consensus is needed in the scientific community to establish an adequate therapeutic algorithm.

Despite the interesting results of our study, the data obtained should be considered with caution due to its limitations. Future prospective studies should be conducted that include control groups and evaluation of psychological variables that may influence the perception of pain.
